# Inguinal Bladder Herniation in the Emergency Department: A Report of Two Cases

**DOI:** 10.5811/cpcem.64458

**Published:** 2026-10-20

**Authors:** Bill Chan, Skyler Sloane, Colleen Campbell, Binh Ly, Rachna Subramony

**Affiliations:** University of California, San Diego, Department of Emergency Medicine, San Diego, California

**Keywords:** Inguinal bladder hernia, hydroureteronephrosis, urosepsis, emergency department, case report

## Abstract

**Introduction:**

Inguinal bladder herniation is a rare subtype of inguinal hernia in which the urinary bladder protrudes into the inguinal canal. It is often asymptomatic but can present with a range of clinical severity in the emergency department (ED).

**Case Report:**

We present a case report of two patients with inguinal bladder hernias. The first case describes a stable patient with bladder herniation without urinary obstruction, successfully managed with outpatient follow-up. The second case illustrates a more severe presentation with bladder involvement causing bilateral hydroureteronephrosis, requiring admission after failed reduction. This patient subsequently re-presented with urosepsis and developed multiple complications, including bacteremia and infective endocarditis.

**Conclusion:**

Inguinal bladder herniation can present with a wide clinical spectrum in the ED. Early recognition, appropriate imaging, and thorough clinical assessment are critical in guiding management and preventing complications.

## INTRODUCTION

We discuss a case report of emergency department (ED) presentations of inguinal bladder hernias, an uncommon subtype of inguinal herniation in which a portion of the urinary bladder protrudes through the inguinal canal. Although inguinal hernias are common, bladder involvement occurs in fewer than 5% of cases, with incidence approaching 10% in older, obese men. Most cases are asymptomatic and discovered incidentally on computed tomography (CT) or intraoperatively. However, these benign presentations have the propensity to progress to significant obstructive uropathy and subsequent urosepsis requiring more aggressive management, making early recognition critical. These cases highlight the variable presentation and urgency of inguinal bladder herniation and emphasize the importance of appropriate escalation of care in the ED. Despite being described in surgical and urologic literature, inguinal bladder herniation remains underrepresented in emergency medicine, making recognition in the acute setting more challenging.

## CASE REPORT

### Case 1

A 72-year-old man with a history of right renal cell carcinoma status postnephrectomy, chronic hernia involving the right side of the bladder, prior hernia repair with mesh, and obesity presented from a skilled nursing facility with worsening abdominal and right flank pain. He stated that he was able to void but had to stand to do so due to the hernia’s position. His vital signs were as follows: heart rate (HR), 87 beats per minute; blood pressure (BP) 126/73 mm Hg; respiratory rate (RR), 18 breaths per minute; and temperature, 97.9 °F. Physical examination revealed a well-appearing patient with suprapubic tenderness without rebound or guarding, and a large nonreducible right inguinoscrotal hernia. Laboratory values revealed no leukocytosis or electrolyte abnormalities. Urinalysis did not show any evidence of infection. Computed tomography of the abdomen and pelvis with contrast demonstrated an inguinal hernia with a 3.9-cm neck containing a portion of the bladder as well as of the small bowel (a new development since the CT of the chronic hernia four months prior) consistent with interval progression of his chronic hernia.

Despite further protrusion of the bladder into the hernia sac, the patient was able to void in the ED, with documented 200-mL urine output. The subsequent postvoid residual measurement was 0 mL, as determined by bladder scan. The patient was provided analgesics and antiemetics in the ED and subsequently discharged back to the skilled nursing facility with anticipated outpatient follow-up with general surgery in three weeks. The patient completed his outpatient elective hernia repair surgery without complication.

### Case 2

A 60-year-old man with no known past medical history presented with acute-onset lower abdominal and left testicular pain beginning the morning of presentation, associated with four episodes of nonbloody, nonbilious emesis. He reported a prior similar episode of inguinal pain and swelling that had self-resolved. He denied trauma, fever, chills, dysuria, hematuria, or genitourinary discharge but noted increasing difficulty with urination. Vital signs were as follows: HR, 62 beats per minute; BP, 149/92 mm Hg; RR, 13 breaths per minute; and temperature, 98 °F. Physical examination demonstrated a soft abdomen with suprapubic and left inguinal tenderness without peritoneal signs. The left inguinal region was markedly swollen with associated bilateral testicular swelling, without overlying erythema or skin changes. Laboratory studies were notable for leukocytosis to 13.4 × 10^9^/L (reference range, 4.0–10.0 × 10^9^/L); no significant electrolyte abnormalities; elevated lactate, 3.6 mmol/L (.5–2.0 mmol/L); and creatinine, 1.27 mg/dL (0.67–1.17 mg/dL).

Computed tomography abdomen and pelvis revealed a large left inguinal hernia containing a significant portion of the anterior bladder, with associated bladder wall thickening and surrounding inflammatory changes (Image 1). Extrinsic compression of the distal ureters had resulted in severe bilateral hydroureteronephrosis, as well as small volume ascites and findings suggestive of inflammatory enteritis (Image 2). Overall, these findings were concerning for clinically significant urinary obstruction secondary to bladder herniation. Throughout his ED course, the patient continued to endorse difficulty with voiding, with only minor dribbling occurring and no ability to create a stream.

Multiple attmpts by general surgery to manually reduce the hernia at bedside were unsuccessful despite manual pressure with Trendelenburg positioning and analgesia. A Foley catheter was successfully placed, yielding 800 mL of output, and the patient was admitted for further management. The urinalysis was negative for infection, and repeat lactate improved to 2.6 mmol/L. The patient remained hemodynamically stable and was discharged the next day after catheter removal, hernia reduction by catheter-induced bladder drainage and manual reduction, and a successful voiding trial.

*CPC-EM Capsule*

What do we already know about this clinical entity?

*Inguinal bladder herniation is rare, often asymptomatic, and usually identified incidentally but can progress to significant obstructive uropathy.*

What makes this presentation of disease reportable?

*These two cases demonstrate the same pathology spanning stable outpatient herniation to obstruction, hydroureteronephrosis, and urosepsis.*

What is the major learning point?

*Inguinal bladder herniation has a broad clinical spectrum. Ultrasound and computed tomography (CT) findings of hydronephrosis or obstruction should prompt urgent evaluation.*

How might this improve emergency medicine practice?

*Greater awareness of this underrecognized entity may prompt earlier ultrasound/CT evaluation and closer attention to voiding symptoms, helping prevent missed obstruction.*

Twelve days after his initial presentation, the patient returned to the ED with fevers, chills, and diarrhea for two days. He denied nausea, vomiting, respiratory symptoms, or sick contacts but experienced decreased urinary output without dysuria or pyuria. He had no pain associated with his groin or chronic left inguinal hernia. Vitals were notable for the following: HR, 124 beats per minute; BP, 140/93 mm Hg; RR, 16 breaths per minute; and temperature, 102.3 °F. Laboratory evaluation was notable for leukocytosis, 19.6 × 10^9^/L (reference range, 4.0–10.0 × 10^9/L); hyponatremia, 126 mmol/L (136–145 mmol/L); and elevated creatinine, 1.27 mg/dL, up from a baseline of 0.7 mg/dL. The initial lactate was 2.4 mmol/L. Urinalysis showed white blood cells, positive leukocyte esterase, and hematuria, concerning for urinary tract infection. The patient was then started on broad-spectrum antibiotics with linezolid and piperacillin-tazobactam and fluid resuscitation.

Given his recent history of bladder-containing inguinal hernia with associated ureteral compression, the clinical picture was concerning for sepsis with a likely urinary source, possibly related to impaired urinary drainage and prior hydroureteronephrosis. Point-of-care ultrasound (POCUS) of the kidneys, bladder, and testicles revealed severe hydronephrosis secondary to the inguinal bladder herniation and persistent herniation of the bladder into the inguinal canal (Image 3). The general surgery service was consulted and confirmed that the hernia remained reducible without indication for acute operative intervention during this presentation. The urology serice recommended Foley catheter placement, which yielded approximately 500 mL of urine output. The patient was admitted to medicine for further management of suspected urosepsis and close monitoring. This progression suggests that even reducible hernias may predispose patients to delayed infectious complications.

The patient’s hospital course was complicated by sepsis with *Staphylococcus epidermidis* both in urine and blood cultures, with a resultant small aortic valve vegetation consistent with infective endocarditis. Despite these complications, the patient improved clinically with antibiotics and supportive care. The leukocytosis and fever resolved, and renal function normalized. He was discharged after a 16-day admission with a Foley catheter in place with close outpatient follow-up, awaiting resolution of endocarditis before proceeding with surgical repair of the hernia.

## DISCUSSION

Inguinal bladder hernias, while rare, are important to consider in patients presenting with flank pain and lower urinary tract symptoms, particularly in the setting of known inguinal hernias. Although many cases are asymptomatic and discovered incidentally, symptomatic presentations may include flank pain, two-stage voiding, urinary retention, and hematuria. When hydronephrosis is encountered on POCUS in a patient with inguinal hernia, further investigation is warranted to determine bladder involvement in herniation, and laboratory evaluation should be performed to assess renal function. More extensive involvement can result in complete bladder herniation, or scrotal cystocele, which is associated with complications such as obstructive uropathy, bowel involvement, and rarely, bladder ischemia.

This case report of two cases highlights the broad clinical spectrum of inguinal bladder herniation. The first case demonstrates a stable presentation without urinary obstruction or infection, allowing for safe outpatient management. By contrast, the second case illustrates a more severe presentation with bladder involvement causing ureteral compression and bilateral hydroureteronephrosis, requiring hospital admission after failed bedside reduction. Together, these cases emphasize that similar anatomical pathology may present with markedly different clinical severity.

Computed tomography remains the diagnostic modality of choice, enabling identification of bladder involvement and associated complications such as hydronephrosis or bowel herniation. Point-of-care ultrasound serves as a valuable adjunct, allowing rapid bedside assessment of bladder volume, urinary retention, and hydronephrosis, and may suggest bladder displacement into the inguinal canal. Point-of-care ultrasound is also useful for identification of bowel obstruction within hernia contents, which we did not see in these cases. When findings on ultrasound are consistent with bladder obstruction, rapid treatment with Foley catheter drainage of the bladder should be considered. Integration of imaging with clinical assessment—including voiding ability and signs of infection or obstruction—guides management and disposition. While stable patients may be managed in the outpatient setting, those with obstruction, infection, or failed reduction require prompt surgical consultation. Early recognition of this condition can directly influence disposition and help prevent downstream complications.

## CONCLUSION

Inguinal bladder herniation is a rare but important diagnosis in the ED, with presentations ranging from incidental findings to obstructive uropathy and sepsis. These cases highlight the importance of integrating imaging findings with clinical assessment, particularly in the case of evaluating urinary function and signs of infection or obstruction. Early recognition and appropriate disposition can prevent significant morbidity associated with delayed diagnosis.

## Figures and Tables

**Image 1:**
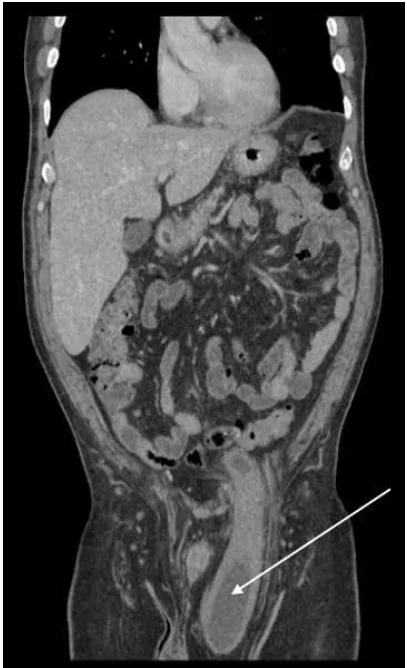
Computed tomography demonstrating inguinal bladder herniation (arrow).

**Image 2:**
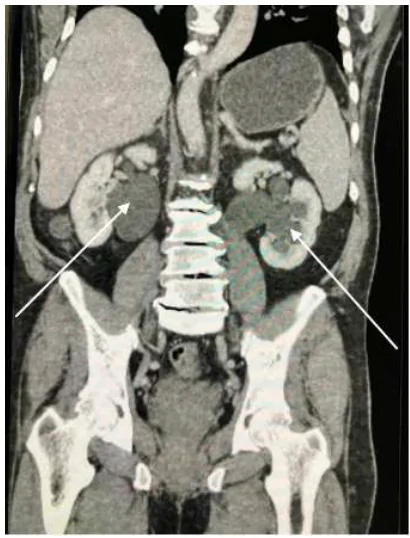
Computed tomography showing bilateral severe hydronephrosis, as indicated by both arrows.

**Image 3:**
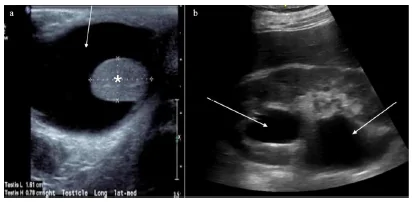
***A***, Ultrasound of bladder herniation into the inguinal canal seen as the anechoic space behind the testicle (the testicle is annotated by the asterisk). The bladder is annotated by the arrow. **B**, Ultrasound of the right kidney demonstrating severe hydronephrosis (arrows).
